# Tardive Dyskinesia in a Patient With Intellectual Disabilities; Diagnostic Challenges and Management Lessons From a Case Study

**DOI:** 10.1192/bjo.2026.11860

**Published:** 2026-06-30

**Authors:** Gabriel Michael, Larteque Lawson

**Affiliations:** Bradford District Care NHS Foundation Trust, Bradford, United Kingdom

## Abstract

**Aims:**

Tardive Dyskinesia (TD) is a movement disorder that typically occurs in individuals with longstanding use of dopamine receptor blocking agents; this typically includes second generation anti-psychotics, but can also include anti-emetic/anti-vertigo drugs and even other drugs. The onset of TD is often insidious with the first symptoms presenting over days to weeks and usually even months, prior to development into a full syndrome. It can only occur 3 months after the exposure to such agents for those under the age of 60 and 1 month of exposure for those aged above 60.

**Methods:**

We present the case of a 66-year-old gentleman with a severe intellectual disability and a history of challenging behaviour. He has been previously under the care of his community learning disability service for a previous history of challenging behaviour but was re-referred on this occasion with the concerns about strange behaviour.

Staff noted and reported a intermittent abnormalities in movement and this gentleman’s case was referred to Psychiatry for follow up. These abnormalities also included difficulties in swallowing food. As part of the psychiatric evaluation the physical health of the gentleman was reviewed and the nature of the intermittent abnormalities in movement prompted a referral to the local Neurology department in order to evaluate whether there may be a form of epilepsy that this gentleman was suffering from. This gentleman was diagnosed with Tardive Dyskinesia after extensive ruling out of potential physical and mental health concerns. His treatment of Tetrabenazine was initiated and has since been tapered up to a total daily dose of 125mg per day. His symptoms have improved and with the aid of his local speech and language therapy team and dietetics team, he is able to safely eat food.

**Results:**

This case highlights the complexities of Tardive Dyskinesia and how these symptoms can be easily overlooked in someone with an intellectual disability. It also underscores the importance of proactive monitoring as well as regular medication review. This case further highlights the importance of a multidisciplinary approach towards the care of individuals with intellectual disabilities.

**Conclusion:**

This case further highlights the need for structures screening for Tardive Dyskinesia in the ID population; particularly those receiving long-term antipsychotics. This case further lends weight to the regular use of movement assessments and reinforces the need for continued deprescribing where clinically appropriate.

